# Disconnected Pancreatic Duct Syndrome: A Rare Complication of Acute Pancreatitis

**DOI:** 10.7759/cureus.103729

**Published:** 2026-02-16

**Authors:** Antonio F Duarte, Ana André, Carlos M Trindade, Alexandra Ferreira, Joao Carapinha

**Affiliations:** 1 Department of General Surgery, Unidade Local de Saúde da Arrábida, Setúbal, PRT

**Keywords:** abdominal sepsis, acute necrotizing pancreatitis, disconnected pancreatic duct syndrome, pancreatitis, step-up approach, surgery

## Abstract

Disconnected pancreatic duct syndrome (DPDS) is a rare but serious complication of necrotizing pancreatitis, characterized by disruption of the main pancreatic duct, which separates a viable upstream pancreatic segment from the downstream ductal system, resulting in persistent pancreatic secretions and recurrent fluid collections. We report a clinical case of acute biliary pancreatitis complicated by DPDS to highlight diagnostic and therapeutic challenges. The patient initially developed necrotizing changes with peripancreatic collections requiring surgical intervention for hemorrhagic control and retroperitoneal drainage, followed by management of postoperative infectious complications and definitive treatment of the biliary etiology. Persistent external pancreatic drainage and intolerance to oral feeding prompted further evaluation, and magnetic resonance cholangiopancreatography demonstrated a complete ductal discontinuity consistent with DPDS. Despite initial clinical improvement, the patient developed recurrent symptoms related to a new intra-abdominal fluid collection, causing gastrointestinal compression. Endoscopic transgastric drainage was successfully performed, leading to the resolution of symptoms and clinical stabilization.

This case illustrates the complex and protracted course that DPDS may entail, as well as the importance of maintaining a high index of suspicion in patients with persistent or recurrent pancreatic collections after severe pancreatitis. Timely imaging, accurate diagnosis, and a multidisciplinary approach integrating surgical, radiologic, and endoscopic expertise are crucial to optimize management and improve outcomes in this challenging condition.

## Introduction

Disconnected pancreatic duct syndrome (DPDS) is a rare and severe complication of acute necrotizing pancreatitis characterized by the total loss of continuity in the main pancreatic duct. This disruption isolates a segment of viable upstream pancreatic tissue from the digestive tract, preventing the drainage of pancreatic secretions [1,2]. As the disconnected pancreatic segment remains functional, continuous exocrine secretion leads to persistent pancreatic fluid collections, external pancreatic fistulae, and recurrent inflammation, contributing to prolonged morbidity and complex clinical courses [3,4]. 

The reported incidence of DPDS varies widely, occurring in up to 10-30% of patients with necrotizing pancreatitis, although it is likely underdiagnosed due to delayed recognition and heterogeneous diagnostic criteria [1,3]. Diagnosis relies on high-quality cross-sectional imaging and ductal evaluation, most commonly magnetic resonance cholangiopancreatography (MRCP), endoscopic retrograde cholangiopancreatography (ERCP), or endoscopic ultrasound (EUS), to confirm complete ductal disruption and identify viable upstream pancreatic tissue [4,5].

Management strategies for DPDS remain controversial and are not standardized, encompassing endoscopic, surgical, or hybrid approaches within a step-up framework tailored to patient-specific factors and institutional expertise, drawing on evidence from both DPDS-specific series and broader studies of necrotizing pancreatitis management [4,6]. According to Maatman et al., endoscopic transmural drainage has emerged as an effective, minimally invasive alternative for selected patients. It offers outcomes comparable to surgery regarding morbidity and mortality, though long-term challenges like recurrence and stent dependency remain [6]. Given its association with recurrent interventions, prolonged hospitalization, and significant healthcare burden, early recognition of DPDS and multidisciplinary management are essential to optimize outcomes [4,6].

We present a clinical case of a woman with disconnected pancreatic duct syndrome following necrotizing acute pancreatitis, highlighting the challenges encountered in disease management and summarizing the key principles of its therapeutic approach.

## Case presentation

We report the case of a 27-year-old woman with a medical history of class I obesity, ultrasound-documented cholelithiasis, and active smoking. She presented to the emergency department with a 6-hour history of epigastric abdominal pain radiating to the back in a belt-like pattern, associated with nausea and vomiting.

Laboratory evaluation revealed cholestatic liver enzyme abnormalities and elevated pancreatic enzymes, with aspartate transaminase at 403 U/L (reference range 5-30 U/L), alanine transaminase at 324 U/L (reference range 4-36 U/L), gamma-glutamyl transferase at 365 U/L (reference range 6-50 U/L), alkaline phosphatase at 159 U/L (reference range 30-120 U/L), amylase at 3209 U/L (reference range 30-110 U/L), and lipase at 5906 U/L (reference range 10-60 U/L). Contrast-enhanced computed tomography (CT) confirmed the diagnosis of mild, uncomplicated acute biliary pancreatitis according to the revised Atlanta classification [7], with two Ranson criteria (leukocytosis and elevated aspartate aminotransferase) [8]. She was admitted for clinical monitoring, intravenous hydration, and analgesia.

On hospital day 2, inflammatory markers worsened with persistence of symptoms. Repeat abdominal CT demonstrated progression of edematous acute pancreatitis without evidence of necrosis or hemorrhage. On hospital day 14, due to further clinical and analytical deterioration, a new CT scan revealed peripancreatic exudates with areas suggestive of necrosis or intralesional hemorrhage, consistent with necrotizing hemorrhagic pancreatitis. The patient underwent emergent laparotomy (or video-assisted retroperitoneal debridement) for surgical hemostasis, retroperitoneal lavage, and drainage, including the placement of a dedicated retroperitoneal drain.

Postoperatively, the patient had persistent turbid drainage, abdominal pain, vomiting, and feeding intolerance. On hospital day 33, she developed a surgical site infection requiring wound exploration, which revealed deep fascial dehiscence with bowel interposition and omental evisceration. Abscess drainage and retroperitoneal lavage were performed, followed by negative-pressure wound therapy. Delayed abdominal wall closure with an interfascial absorbable mesh (Vicryl) was completed on hospital day 36, with definitive wound closure on day 40.

Due to persistent upper abdominal pain, a laparoscopic cholecystectomy was performed on hospital day 59. Despite multiple courses of antibiotics, the patient’s clinical course was complicated by a refractory surgical site infection (SSI), persistent abdominal pain, and high-output drainage of necrotic pancreatic debris. Additionally, ongoing feeding intolerance necessitated the initiation of total parenteral nutrition (TPN). Due to the persistence of high-amylase drain output despite weeks of conservative management, an MRCP was performed on hospital day 98. The imaging revealed a mid-duct disruption of the Wirsung duct, confirming the diagnosis of DPDS.

**Figure 1:**
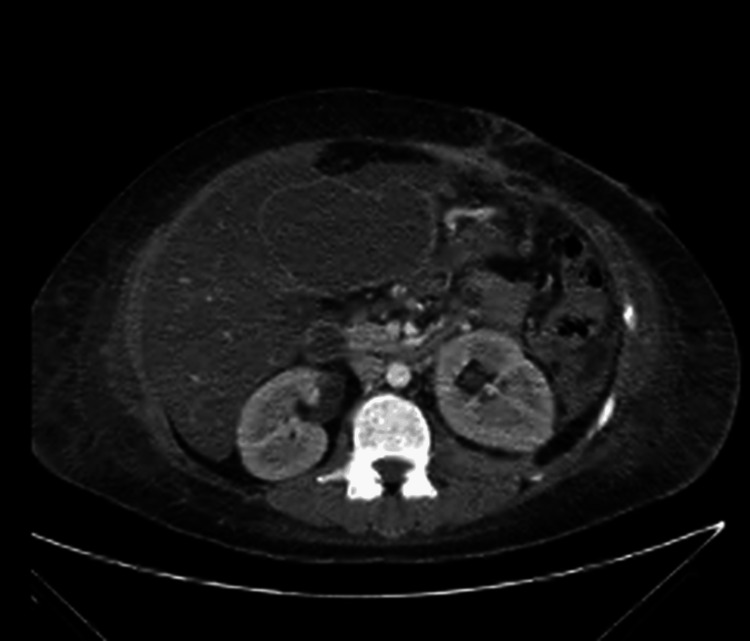
Subcapsular hepatic collection Collection adjacent to the inferior hepatic margin with mass effect on adjacent structures

On hospital day 124, a sudden increase in drain output was accompanied by respiratory distress. CT imaging revealed a large newly formed collection adjacent to the inferior hepatic margin with mass effect on adjacent structures, as well as significant ascites (Figure 1).

**Figure 2:**
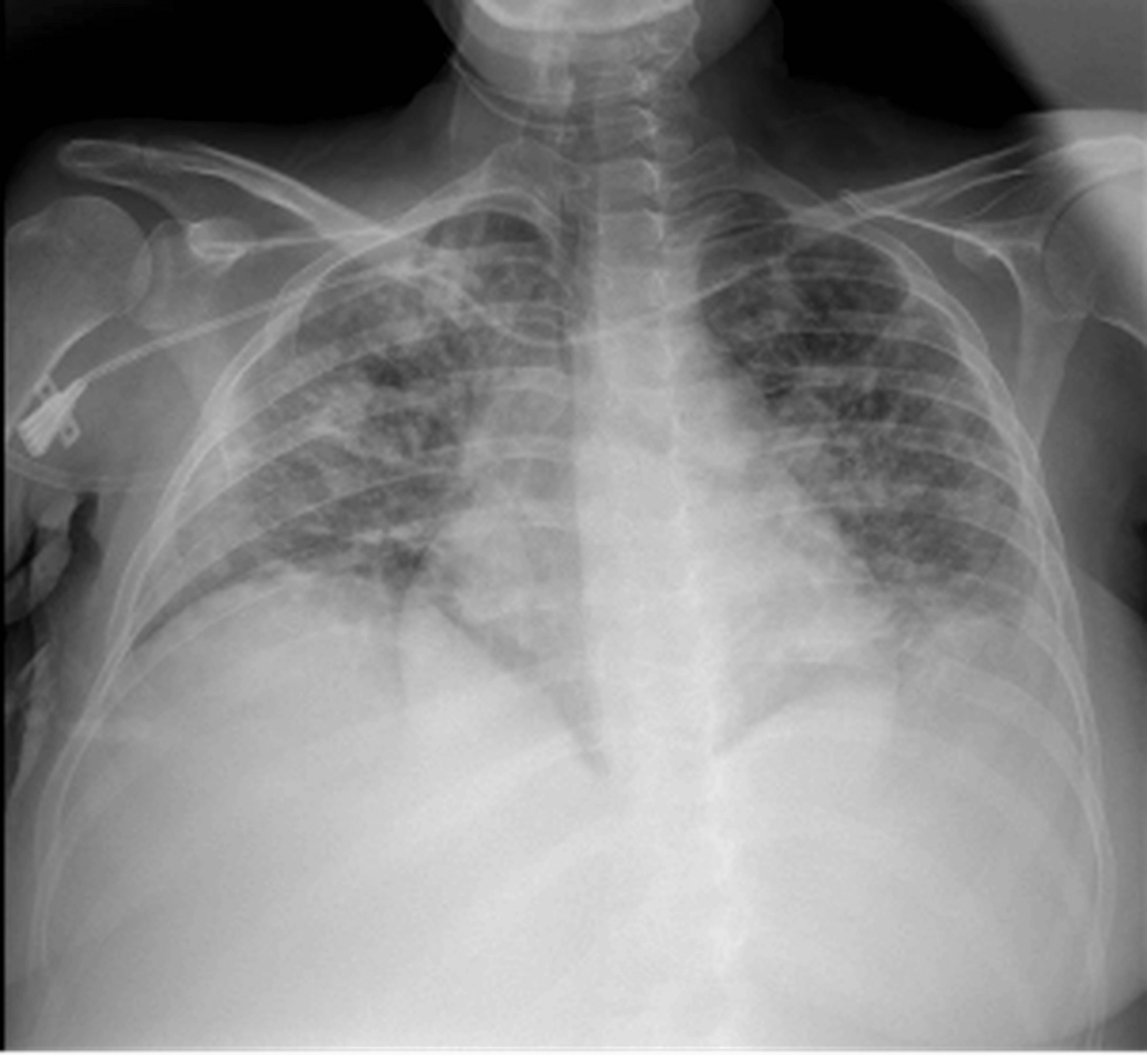
Imageological aspects of ARDS Thoracic X-Ray findings consistent with the diagnosis of ARDS, specifically bilateral diffuse alveolar opacities, with rapid progression. ARDS: Acute Respiratory Distress Syndrome

Therapeutic paracentesis yielded pancreatic fluid with an amylase level of 1,517 U/L. The patient subsequently developed acute respiratory distress syndrome (ARDS) and required admission to the intensive care unit with non-invasive ventilation for four days (Figure 2).

Following respiratory improvement, she returned to the surgical ward, where progressive clinical improvement was noted, including a reduction in abdominal pain, decreasing drain output, and gradual advancement of oral intake. Multidisciplinary evaluation at a tertiary referral center supported medical discharge with proton pump inhibitor therapy (pantoprazole 40 mg twice daily) and pancreatic enzyme supplementation, with outpatient follow-up. She was discharged on hospital day 204.

**Figure 3:**
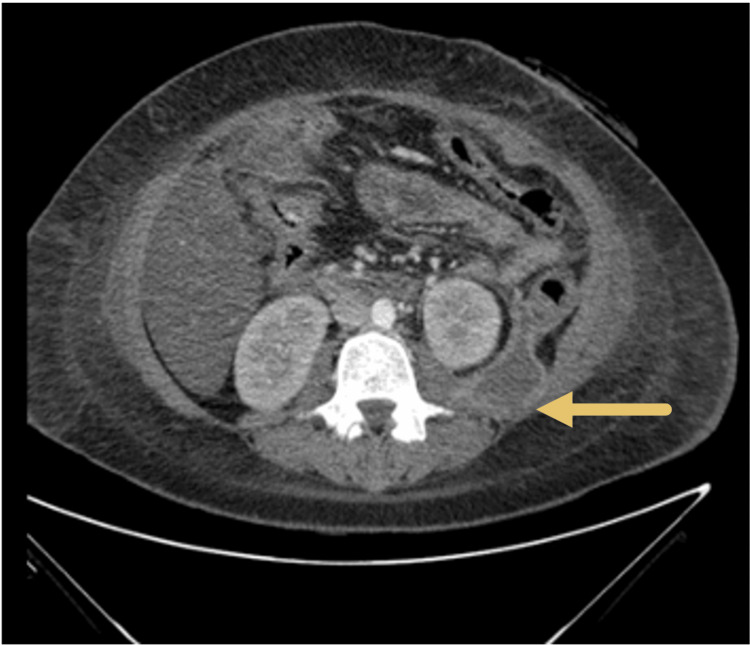
Left pararenal fluid collection (arrow pointing for the collection) Left pararenal fluid collection causing duodenal compression

One month later, the patient re-presented with epigastric pain, vomiting, and feeding intolerance. CT imaging demonstrated a large left pararenal fluid collection causing duodenal compression (Figure 3).

She was referred to a specialized center, where endoscopic transgastric drainage with stent placement was successfully performed. The patient showed rapid clinical improvement and was discharged 48 hours after admission. The stent was removed six months later. At follow-up, the patient remained asymptomatic, with progressive weight gain and no further complications.

## Discussion

Disconnected pancreatic duct syndrome represents a unique and challenging complication of necrotizing acute pancreatitis, characterized by functional separation of the upstream pancreatic parenchyma from the gastrointestinal tract due to complete disruption of the main pancreatic duct. As highlighted in our patient, this condition leads to persistent pancreatic fluid collections, external fistulas, and recurrent inflammation, reflecting the pathophysiological consequences of a functioning but anatomically isolated pancreatic segment [1,3].

The incidence of DPDS has been variably reported, with estimates ranging from 10-30% of patients with necrotizing pancreatitis [1,3], consistent with the occurrence in our patient, who developed mid-duct disruption following hemorrhagic necrotizing pancreatitis of biliary etiology. Prior studies, such as those by Gámez-del-Castillo et al., emphasize that DPDS requires both necrosis of the main pancreatic duct and preservation of upstream parenchymal function [2]. These criteria were met in our patient, whose viable pancreatic tissue continued exocrine secretion, precipitating recurrent collections and high-output drainage [2,3]. Extensive parenchymal necrosis precludes DPDS, underscoring the importance of assessing both ductal integrity and tissue viability [1,3].

Clinically, DPDS often evolves through a biphasic course. Initially, patients present with typical manifestations of necrotizing pancreatitis, including systemic inflammatory response and organ dysfunction, as seen in our patient during the first weeks of hospitalization. Subsequently, features specific to DPDS, such as persistent or recurrent collections, high-output pancreatic fistulae, chronic abdominal pain, and complications such as infection or hemorrhage, are observed [4,6]. In our case, the protracted postoperative course, including refractory surgical site infections, feeding intolerance, and recurrent fluid collections, exemplifies the severe morbidity associated with delayed recognition of DPDS. This aligns with prior reports suggesting that delayed diagnosis significantly prolongs hospitalization and increases the risk of invasive interventions [4,6].

Diagnostic imaging plays a critical role in confirming DPDS. While contrast-enhanced CT is commonly used for initial evaluation, it may not reliably distinguish true ductal disruption from compressive effects of collections or edema [1]. MRCP or EUS can better delineate ductal anatomy and detect viable upstream parenchyma, as demonstrated in our patient, whose diagnosis was confirmed by MRCP at day 98. Notably, the observation that the pancreatic duct communicated with collections at an acute angle supports prior findings that this configuration is predictive of true ductal disconnection rather than simple compression [1,2]. ERCP remains the gold standard for confirming complete disruption but carries procedural and infectious risks and is generally reserved for cases with therapeutic intent [4].

Management strategies for DPDS remain heterogeneous and require individualized, multidisciplinary planning. Conservative treatment alone is generally insufficient due to ongoing exocrine secretion from the disconnected segment [3,4]. Percutaneous drainage, while useful for acute complications, is associated with high rates of external fistula formation and recurrence. Surgical options aim either to resect the upstream pancreas or establish internal drainage into the gastrointestinal tract. Resection effectively eliminates the source of secretions but risks endocrine and exocrine insufficiency, whereas internal drainage procedures, such as cystogastrostomy or cystojejunostomy, preserve pancreatic function and have been associated with lower rates of postoperative complications, albeit often necessitating reintervention [5,6,9].

Endoscopic management has emerged as a less invasive alternative, with transmural stent placement under endoscopic ultrasound guidance demonstrating efficacy comparable to surgical approaches in select patients [6,9,10]. Our patient’s successful endoscopic transgastric drainage and subsequent stent removal mirror current evidence supporting long-term stent placement as a viable first-line approach for well-defined collections, facilitating symptom resolution while preserving pancreatic parenchymal function. However, as observed in our patient, recurrent collections may necessitate delayed intervention, emphasizing the need for ongoing surveillance and multidisciplinary follow-up.

This case underscores several critical lessons reinforced by prior research. First, early recognition of DPDS is paramount, as delayed diagnosis contributes to prolonged morbidity, repeated interventions, and healthcare resource utilization. Second, high-quality imaging with MRCP or EUS is essential for accurate assessment of ductal integrity and parenchymal viability. Third, management should follow a step-up approach tailored to patient-specific factors, beginning with initial supportive care, followed by minimally invasive drainage when feasible, and surgical intervention reserved for refractory cases. Finally, long-term follow-up is necessary to monitor for recurrence, nutritional compromise, and pancreatic insufficiency.

## Conclusions

DPDS is a serious complication of necrotizing acute pancreatitis, whose diagnosis is often delayed due to a lack of awareness, with consequences for the patient and prolonged hospital stays. The diagnosis is based on imaging studies and should always be considered as a diagnostic possibility in patients with refractory pancreatic collections in the context of acute pancreatitis. Our patient exemplifies the complex natural history of DPDS, from initial necrotizing pancreatitis to delayed recognition and successful endoscopic management. This case reinforces the evolving paradigm favoring early, multidisciplinary evaluation and minimally invasive intervention while highlighting the persistent challenges in diagnosis, recurrent collections, and long-term care.

Surgery has historically been the treatment modality of choice for these patients, ensuring internal drainage of collections. However, with the development of endoscopic and minimally invasive techniques, management of this condition is likely to increasingly transition toward these approaches.
